# Effects of a rollator on fall prevention in Community-Dwelling people with Parkinson’s Disease: A prospective cohort study

**DOI:** 10.1016/j.prdoa.2023.100190

**Published:** 2023-02-22

**Authors:** Kohei Okuyama, Yoshimi Matuo

**Affiliations:** a Department of Physical Therapy, School of Health Science, Bukkyo University, 7 Nishinokyo Higashi-toganoocho, Nakagyo-ku, Kyoto 604-8418, Japan; b Department of Health and Sports Sciences, Mukogawa Women’s University, 6-46 Ikebiraki, Nishinomiya, Hyogo 663-8558, Japan

**Keywords:** Parkinson’s disease, Rollator, Falls

## Abstract

**Introduction:**

This study aimed to investigate the effect of a rollator on the prevention of falls in patients with Parkinson’s disease (PD) during outdoor walks.

**Method:**

This study examined 30 community-dwelling patients with PD. Factors associated with falls were classified into clinical patient background, physical function, and psychophysiological function factors. The number of falls and subsequent injuries was observed over a period of greater than 6 months, if patients were using rollators while falls happened.

**Results:**

Participants who used a rollator had a significantly lower fall rate, number of falls, and injury rate than those who did not use a rollator (p < 0.05).

**Conclusion:**

A rollator could protect patients with PD from falls. Additionally, when considering the use of a rollator for patients with PD, it is important to assess the patient’s physical and psychophysiological functions.

## Highlights

• A rollator is effective in preventing falls for people with Parkinson’s disease.
• A rollator reduces the injury rate in cases of falls.
• Assessment of psychophysiological functions is essential for the use of a rollator.

## 1 Introduction

Patients with Parkinson’s disease (PD) are known to experience gait disturbances, including decreased stride length, walking speed, arm swing, and foot clearance as well as increased gait rhythm variability and stride asymmetry from early stages of the disease [1]. These changes gradually become more pronounced as the disease progresses. Therefore, such patients have a high risk of falling, i.e., the risk is six times higher in patients with PD than in healthy older people [2]. Walking is the most common activity during which falls may occur [3]. Falls can cause trauma, fracture, and—in some cases—even death, and they inevitably result in deterioration in activities of daily living (ADL) and quality of life to a greater extent than disease progression. Additionally, complications due to falls can increase medical costs [4]. Therefore, from the perspective of health economics, falls are an important issue in patients with PD requiring intensive intervention.

Walking aids are commonly used tools to address this issue [5]. Moreover, it has been reported that 30 % of patients with PD use a walking aid during indoor walks, whereas 52 % use it during outdoor walks [6]. The number of people using walking aids has increased over time; in particular, the number of people using a rollator has rapidly increased recently [7]. Thus, many patients with PD use rollators in home healthcare settings. The function of a rollator is to provide stability while standing and walking by expanding the body’s support base [8]; however, studies on the use of rollators in patients with PD are few [9]. The use of laser lights in the U-Step rollator has been reported to result in reductions in the percentage of falls and weekly frequency of falls [10]. However, the reality is that most people use general four-wheel seat rollators and fewer laser light U-Step rollators [7]. After an extensive search, we found that no studies were aimed at determining whether a rollator can prevent or increase the risk of falls in specific gait disorders, such as freezing of gait. Therefore, in this study, we investigated whether a rollator prevents falls in patients with PD during outdoor walks.

## 2 Participants and methods

### 2.1 Participants

Patients with PD (n = 30) who attended nine intractable disease day-care facilities, day rehabilitation services, and home-visit rehabilitation services from three medical groups were included in this study. Patients with PD were defined as those who had been diagnosed by a neurologist per the diagnostic criteria of the International Parkinson and Movement Disorder Society (MDS) [11]. The mean age of the participants was 69.9 ± 6.1 years; of the 30 participants, 13 were males and 17 were females. The duration of PD was 141.6 ± 93.9 months. Using the Hoehn and Yahr scale (H&Y scale), the stage of PD was determined to be Ⅰ, Ⅱ, III, and IV in 2, 2, 15, and 11 participants, respectively. The inclusion criteria were as follows: individuals who consented to participate in the study and those who were able to walk without assistance. Conversely, the exclusion criteria were as follows: individuals with dementia or suspected dementia, as assessed using the Mini-Mental State Examination (MMSE) with a score of ≤ 24; those with neurological or orthopedic problems that might have affected their gait; and those who could not be followed up for 6 months owing to hospitalization or death.

This 6-month prospective cohort study was conducted by three medical groups. Approval for this study was obtained from the Ethics Committee of Mukogawa Women’s University (approval number 13–54). With the help of the research statement, the content and purpose of the study were explained to the participants in writing and orally. The participants provided consent to participate in the study by signing a research participation consent form.

## 3 Methods

The participants were asked whether they used rollator as a walking aid. Rollator users were asked whether they chose to use it because they felt the need for the same or because others recommended it. They were also asked to freely describe how they felt about using a rollator. Rollator nonusers answered with an open-ended question why they did not use a rollator. Rollator was defined as a walking aid consisting of left and right frames and a central tube connecting them that could be used alone, supported by hands or arms, and equipped with wheels at the lower ends of the left and right frames [12]. Factors related to falls [2], [13] were assessed by categorizing them into clinical background, physical function, and psychophysiological function factors. Subsequently, the details of the participants’ falls were assessed for 6 months from the date of examination and measurement. Notably, each examination and measurement was performed during the pharmacological period. Additionally, three experienced physiotherapists (except for the researcher) performed each test and measurement. It was specified that the questions and words should not be paraphrased to ensure that there were no differences between the content of the statements of different examiners.

Clinical background information was obtained based on the following: history of falls, medication status, H&Y scale as an index of PD severity, the Movement Disorder Society-Sponsored Revision of the Unified Parkinson’s Disease Rating Scale (MDS-UPDRS), body mass index (BMI), the Barthel Index (BI), and the Functional Independence Measure (FIM) as an index of ADL. For MDS-UPDRS, the records were measured at the on-time, which was at least 1 h after medication. The subjects were also surveyed for the presence of orthostatic hypotension and dyskinesia using the MDS-UPDRS questionnaire. Regarding medication status, antiparkinsonian drugs were assessed using a conversion table to determine the levodopa equivalent daily dose (LEDD) [14]. The presence or absence of benzodiazepines was also assessed.

Physical function was assessed using the following tests and measurements: the Timed Up and Go Test as a measure of balance, the 10-meter walk test as a measure of walking ability, grip strength, the Freezing of Gait Questionnaire (FOGQ), a hand-held dynamometer (Moby MT-100, Sakai Medical Corporation) to evaluate knee extension muscle strength, and an activity meter (Active Style Pro HJA-350IT, Omron Health Care Co.) to assess physical activity. Physical activity of the participants was measured by informing them to wear an activity meter on the waist for 1 week, except while bathing and sleeping. Participants who answered “at least once a week” to the third question of the FOGQ—“How much does your foot stick to the ground while walking, changing direction, or when starting to walk?”—were defined as having freezing of gait [15].

The psychophysiological functional assessment included five scales. The MMSE, Self-rating Depression Scale (SDS), and Modified Falls Efficacy Scale (MFES) assessed confidence in performing the movement without falling safely. The modified Gait Efficacy Scale (mGES) measured confidence in walking safely, and the Trail Making Test Part A (TMT-A) assessed attention and executive function.

Patients with falls were followed up for 6 months from the date of examination and measurement. On the day the participants visited the facilities, they or their caregivers reported the frequency of falls since the date of previous hospital or facility visits. The data were recorded in their communications books and transferred to their medical records to ensure that the number of falls and injuries caused by falls could be assessed. Falls were defined as contact of the knees or hands with the ground or a lower point, not by one’s own will [16].

The participants were classified into two groups based on whether they used a rollator. The rate, frequency, and injury rate after a fall and clinical patient background, physical function, and psychophysiological function were analyzed using a two-sample *t*-test, the Mann–Whitney test, the χ2 test, and the Fisher’s exact test. In addition, risk ratios and confidence intervals for falls and fractures were determined. Statistical analysis was performed using the IBM SPSS Statistics ver. 26.0 (IBM Corp.) with a significance level of 0.05.

## 4 Results

The survey results revealed that 14 individuals used a rollator, whereas 16 did not. Nine (64 %) used a rollator because they felt that its use was necessary, whereas five (36 %) chose to use it because others recommended it. Regarding the use of rollator, eight (50 %) felt that the use was safe, six (38 %) felt that it was stable (patients were less likely to fall, more comfortable while walking, or demonstrated improved balance), and two (13 %) felt that it was functional (patients were able to carry luggage, sit, and rest). Answers to the open-ended question about why they did not use rollator were as follows: nine (56 %) answered that they did not feel the need; five (31 %) answered that they had functional problems (rollator was getting in the way, patient was not moving fast enough, and patient was not able to move around freely); and four (25 %) answered that they had appearance-related concerns (concern regarding the common use of rollator by older people and regarding others’ perceptions) (duplicate answers were available).

In total, 20 (66.7 %) participants experienced falls; 19 (63.3 %) falls were during indoor walks, 13 (43.3 %) were during outdoor walks, and 12 (40 %) were during both indoor and outdoor walks.

The clinical background of the participants is shown in Table 1. Age (p = 0.254), gender (p = 0.696), duration of illness (p = 0.895), medication status (p = 0), H&Y scale (p = 0.919), BMI (p = 0.100), BI (p = 0.135), FIM (p = 0.756), MDS-UPDRS On (p = 0.393) Off (p = 0.993), history of falls (p = 0.491), and medication status for levodopa (p = 0.995), dopamine agonist (p = 0.993), and benzodiazepine (p = 0.648) were not significantly different between the two groups. Moreover, the ten-meter walk test (p = 0.640), TUGT (p = 0.260), grip strength (p = 0.825), knee extensor strength (p = 0.901), activity level (p = 0.862), FOGQ (p = 0.891), and freezing of gait (p = 0.390) in physical function were not significant different between the two groups (Table 2). MMSE (p = 0.949), SDS (p = 0.304), and mGES (p = 0.102) in psychophysiological function were also not significantly different; however, MFES and TMT were significantly lower in the rollator user group than those in the rollator nonuser group (p < 0.01) (Table 3).

**Table 1:** Comparison of patients’ clinical background between the rollator user and nonuser groups.

	Rollator user group (n = 14)	Rollator nonuser group (n = 16)	p-value	Effect size
Age^†^	70.9 ± 6.5	68.4 ± 5.5	0.254	0.22
Gender (male/female)^‡^	6/8	8/8	0.696	0.07
Duration of PD^†^	139.1 ± 82.2	143.8 ± 105.7	0.895	0.03
H&Y scale^§^	Ⅰ 1/Ⅱ 0/III 6/IV 7/V 0	Ⅰ 1/Ⅱ 1/III 8/IV 6/V 0	0.919	0.20
BMI (kg/m^2^)^†^	19.8 ± 2.1	21.0 ± 2.3	0.100	0.07
BI^†^	73.6 ± 15.8	82.5 ± 15.8	0.135	0.28
FIM^†^	98.1 ± 12.9	95.9 ± 23.4	0.756	0.06
MDS-UPDRS score^†^				
Total	58.0 ± 25.6	48.9 ± 22.1	0.393	0.15
Part III	20.1 ± 8.9	20.3 ± 9.1	0.963	0.01
History of falls (n,%)^‡^	7 (50.0)	10 (62.5)	0.491	0.13
LEDD (mg/day)^†^	482.1 ± 176.4	491.9 ± 221.1	0.905	0.05
Benzodiazepine (+/−)^||^	2/12	2/14	0.648	0.03
Orthostatic hypotension (+/−)^||^	7/7	7/9	0.732	0.06
Dyskinesia (+/−)^||^	5/9	5/11	0.550	0.04

Mean ± standard deviation, PD: Parkinson’s Disease, H&Y scale: Hoehn and Yahr scale, BMI: Body Mass Index, FIM: Functional Independence Measure, MDS-UPDRS: Movement Disorder Society-Sponsored Revision of the Uniﬁed Parkinson’s Disease Rating Scale, LEDD: levodopa equivalent daily dose.

†: two-sample *t*-test/r, ‡: χ2 test/φ, §: Fisher’s exact test/Cramer’s V, ||: Fisher’s exact test/φ.

**Table 2:** Comparison of physical function between the rollator user and nonuser groups.

	Rollator user group (n = 14)	Rollator nonuser group (n = 16)	p-value	Effect size
10-meter walk test (seconds)^†^	12.5 ± 2.7	11.7 ± 3.2	0.640	0.14
TUGT (seconds)^†^	18.6 ± 4.1	14.2 ± 7.9	0.260	0.28
Grip strength (kg)^†^	19.4 ± 7.5	20.1 ± 7.8	0.825	0.05
Knee extension muscle strength (kg)^†^	13.4 ± 8.2	13.7 ± 5.3	0.901	0.03
Activity amount (step)^‡^	1,003 (910–2153)	1,006 (760–1751)	0.862	0.03
FOGQ (point)^†^	10.4 ± 3.6	10.7 ± 5.0	0.891	0.03
Freezing of gate (+/−)^§^	10/4	9/7	0.390	0.16

Mean ± standard deviation, Median (25 percentile–75 percentile), TUGT: Time Up And Go Test, FOGQ: Freezing of Gait Questionnaire.

†: two-sample *t*-test/r, ‡: Mann–Whitney test/r, §: χ2 test/φ.

**Table 3:** Comparison of psychological function between the rollator user and nonuser groups.

	Rollator user group(n = 14)	Rollator nonuser group(n = 16)	p-value	Effect size
MFES (point)^†^	57.0 ± 27.7	94.3 ± 33.9	0.003**	0.53
TMT-A (second)^‡^	100.0 (57.2–142.4)	136.5 (100.0–227.8)	0.008**	0.48
MMSE (point)^†^	27.1 ± 2.0	27.1 ± 1.9	0.949	0.01
SDS (point)^†^	33.5 ± 4.7	30.9 ± 6.3	0.304	0.24
MGES (point)^†^	49.3 ± 17.3	64.3 ± 22.2	0.102	0.37

Mean ± standard deviation, Median (25 percentile–75 percentile), MFES: Modified Falls Efficacy Scale, TMT-A: Trail Making Test Part A, MMSE: Mini-Mental State Examination, SDS: Self-rating Depression Scale, MGES: Modified Gait Efficacy Scale.

†: two-sample *t*-test/r, ‡: Mann–Whitney test/r, **: p < 0.01.

A comparison of the fall rate, number of falls, and injury rate after falls between the two groups is summarized in Table 4. The fall rate for indoor walks was not significantly different (p = 0.510); however, it was significantly lower for outdoor walks in the rollator user group (p < 0.05), with a risk ratio of 0.477 (95 % confidence interval: 0.240–0.951). Similarly, there was no significant difference in the number of falls during indoor walks (p = 0.556); however, the number of falls during outdoor walks was significantly lower in the rollator user group (p < 0.05). The comparison of injury rates after falls revealed that the rollator user group had a significantly lower rate of falls (p < 0.05), with a risk ratio of 0.510 (95 % confidence interval: 0.281–0.926). Notably, only two rollator users (14 %) had knee injuries. In contrast, among rollator nonusers, seven had injuries in the knee (43 %), three in the buttock (19 %), two in the forearm (13 %), two in the face (13 %), one in the lower back (6 %), and one in the head (6 %). One (6 %) had a clavicle fracture (duplicate answers were available).

**Table 4:** Comparison of fall rate, number of falls, and injury rate after falls between the rollator user and nonuser groups.

		Rollator user group (n = 14)	Rollator nonuser group (n = 16)	p-value	Relative risk (95 % CI)	Effect size
Fall rate (n,%)†	Outdoor	3 (21.4)	10 (62.5)	0.024*	0.477 (0.240–0.951)	0.41
	Indoor	8 (57.1)	11 (68.8)	0.510	0.729 (0.283–1.877)	0.12
Number of falls (times/six months)^‡^	Outdoor	0 (0–1.0)	6.0 (0–21.8)	0.012*		0.46
	Indoor	3.5 (0–30.8)	4.0 (0–72.0)	0.556		0.11
Injury rate after falls (n,%)	Bruise/abrasion†	2 (14.3)	9 (56.3)	0.017*	0.510 (0.281–0.926)	0.43
	Fracture^§^	0 (0)	1 (6.3)	0.533	0.938 (0.826–1.064)	0.17

Median (25 percentile –75 percentile), CI: Confidence Interval.

†: χ2 test/φ, ‡: Mann–Whitney test / r, §: Fisher's exact test/φ, *: p < 0.05.

## 5 Discussion

In this study, 47 % community-dwelling patients with PD used a rollator. In the living environment in Japan, a rollator is usually used outdoors and rarely indoors. Notably, the participants in this study only used the rollator outdoors.

The fall rate of the participants was 67 %, which is similar to that of a previous study of patients with PD, with a fall frequency of 40 %–70 % [17]. Thus, the risk of falling was the same for both the study participants and the general population with PD. Bloem et al. [2] reported that falls in patients with PD are more likely to occur indoors. In this study, the indoor fall rate was very high (63 %); however, the outdoor fall rate was also high (43 %). This is because subjects for day rehabilitation services were relatively active. The number of falls and the rates of falls and injuries was lower in the group using rollator outdoors, indicating that rollator use is associated with fall prevention. Additionally, rollator users had no injuries on their face or head and no fractures. This finding suggests that even if they fall accidentally, the possibility of a serious accident is lower. A study by the Swedish Welfare Research Institute [18] reported that rollator use could reduce the risk of fractures caused by falls, even in people with poor health. This study found the same effect in patients with PD. Moreover, knees were the most commonly injured body part. It has been reported that 46 % of falls in patients with PD are forward falls [19]; this finding was also supported by the present study.

The patients in this study are enrolled in daycare facilities, day rehabilitation services, and home-visit rehabilitation services. It has been demonstrated that institutionalization is considered a milestone of disease progression in PD, and in some way, falls (another disease milestone) and institutionalization could be two different factors co-contributing to the advanced stage of PD [20]. Therefore, it is important to prevent falls, and a rollator is most likely useful in preventing falls in patients with late-stage PD.

There were no differences in patients’ clinical background in terms of age, gender, medication status, PD severity, ADL, and the presence of dyskinesia and orthostatic hypotension indicating a similar risk of falls in both groups.

Orthostatic hypotension is present in 30–60 % of PD [21], and 47 % of patients in this study had orthostatic hypotension. Also, the presence of orthostatic hypotension could be another contributing factor for falls and for the advanced PD stage [22]. It is not clear whether orthostatic hypotension influenced the occurrence of falls in this study; however, the use of the rollator prevented falls.

Physical function was not significantly different between the two groups. The purpose of rollator use was hypothesized to compensate for the reduction in lower extremity muscle strength and walking ability. However, this study found no significant differences in physical function between the two groups. Therefore, it has been suggested that patients with PD can use rollators regardless of the degree of gait freezing, lower extremity muscle strength, or the severity of PD. Although PD is known to have freezing of gait as a risk factor for falls, our finding that patients with freezing of gait can use a rollator constitutes important evidence.

Regarding psychophysiological function, the rollator user group had a greater fear of falling and better attention and executive function than the rollator nonuser group. Previous studies [2], [23] have reported that increased fear of falling in patients with PD leads to decreased activity as well as activity avoidance; however, in the present study, the activity volume was equivalent between the two groups, and there was no decrease in activity, suggesting that the fear of falling leads to rollator use in order to maintain the activity. Furthermore, based on the survey of rollator users, 86 % answered that the reason for using rollator was safety and stability, indicating that the fear of falling led to the choice of rollator.

In contrast, there was no significant difference in the rate and number of indoor falls between the two groups, suggesting that the two groups had the same likelihood of falling. However, some patients with PD did not use rollator. Therefore, nonusers of rollator were also examined in this study. In the rollator nonuser group, the fear of falling as well as attention and executive function decreased. In healthy older adults, it has been reported that the fear of falling is felt more strongly by those who fall frequently [24]. Nakazono et al. [25] found that patients with PD who fall frequently (more than once a week) are less afraid of falling. In the survey of rollator nonusers in the present study, more than 50 % of the participants responded that although they experienced multiple falls, they did not feel the need to use rollator, consistent with the results of previous studies.

In older people, attention and executive function has been reported to be associated with falls [26]. In addition, Diddle et al. [27] reported that a decline in attention and executive function in patients with PD contributes to falls. Walking is usually unconscious and possible even when the attention is on performing dual tasks, such as having conversations [28]. However, patients with PD have impaired automatism because of damage to the basal ganglia, and in such patients, walking must be performed with attention. Additionally, it has been reported that patients with PD prioritize other tasks instead of walking [29], and compared with healthy older people, doing double duty reduces the ability to walk in patients with PD [30].

The present study suggests that attention to falls, rather than physical function, such as the degree of gait freezing, determines rollator use. Patients with PD are more likely to be distracted by the task or environment; therefore, they need better attention and executive function to use a rollator. In contrast, it has been suggested that people with poor psychophysiological functioning have difficulty using a rollator. Therefore, it is important to evaluate physical and psychophysiological functions when considering the use of rollator.

The first limitation of this study is that the study was conducted at only three institutions, and the number of cases was limited. It is therefore uncertain whether the results accurately reflect the population. Second, multivariate analysis could not be performed owing to the small number of cases; thus, the influence of other factors could not be eliminated. Third, the impact of the rollator models on the results was not analyzed. So, it is unclear which is more fall-protective than a U-step rollator. Finally, it was found that the severity of PD was high among participants; therefore, it is unclear whether similar results could be obtained in a population with a lower PD severity. Further studies are required to include more cases and various PD severities in many facilities.

## 6 Conclusion

This study examined the effect of rollator on preventing falls in patients with PD during outdoor walks. It was found that the fall rate, number of falls, and injury rate after a fall were lower in those using rollator. Therefore, it was suggested that the use of a rollator could prevent falls.

The patients’ clinical background and physical function were not significantly different between the two groups. Regarding psychophysiological function, the rollator user group had a greater fear of falling and better attention and executive function.

This study suggests that attention to falls, rather than physical function, such as the degree of gait freezing, determines rollator use. Patients with PD are more likely to be distracted by the task or the environment; therefore, they need better attention and executive function to use rollator.

In contrast, it has been suggested that people with poor psychophysiological functioning have difficulty using rollator. Therefore, it is important to evaluate physical and psychophysiological functions when considering the use of rollator.

In conclusion, this study provides important evidence for the fall-preventive effects of rollators in community-dwelling patients with PD and suggests that better attention and executive functions are required for adaptation to a rollator.

## Declaration of Competing Interest

The authors declare that they have no known competing financial interests or personal relationships that could have appeared to influence the work reported in this paper.

## References

[1] Mirelman A., Bonato P., Camicioli R., Ellis T.D., Giladi N., Hamilton J.L., Hass C.J., Hausdorff J.M., Pelosin E., Almeida Q.J. (2019). Gait impairments in Parkinson’s disease. Lancet Neurol..

[2] Bloem B.R., Grimbergen Y.A., Cramer M., Willemsen M., Zwinderman A.H. (2001). Prospective assessment of falls in Parkinson’s disease. J. Neurol..

[3] Koura A., Takashima C., Uchiyama M., Matsuo Y., Abe K. (2005). Falls in patients whit Parkinson’s disease: a questionnaire investigation. JAOT.

[4] Pressley J.C., Louis E.D., Tang M.X., Cote L., Cohen P.D., Glied S., Mayeux R. (2003). The impact of comorbid disease and injuries on resource use and expenditures in parkinsonism. Neurology.

[5] Constantinescu R., Leonard C., Deeley C., Kurlan R. (2007). Assistive devices for gait in Parkinson’s disease. Parkinsonism Relat. Disord..

[6] Kader M., Jonasson S.B., Iwarsson S., Odin P., Nilsson M.H. (2018). Mobility devices use in people with Parkinson’s disease: a 3-year follow-up study. Acta Neurol. Scand..

[7] Ministry of Health, Labour and Welfare, Statistics on long: term care benefit expenses (formerly Survey on Long: term Care Benefit Expenses). https://www.mhlw.go.jp/toukei/list/45-1.html (Available from: April 23, 2022).

[8] Yoshimura S., Hasegawa M. (2000). Application criteria of walking aids. Jpn. J. Phys. Ther..

[9] García-Bustillo Á., Valiñas-Sieiro F., Allende-Río M., González-Santos J., Cubo E. (2022). and the Multidisciplinary Telemedicine Group, Assistive devices for personal mobility in Parkinson’s disease: A systematic review of the literature. Mov. Disord. Clin. Pract..

[10] Donovan S., Lim C., Diaz N., Browner N., Rose P., Sudarsky L.R., Tarsy D., Fahn S., Simon D.K. (2011). Laserlight cues for gait freezing in Parkinson’s disease: an open-label study. Parkinsonism Relat. Disord..

[11] Postuma R.B., Berg D., Stern M., Poewe W., Olanow C.W., Oertel W., Obeso J., Marek K., Litvan I., Lang A.E., Halliday G., Goetz C.G., Gasser T., Dubois B., Chan P., Bloem B.R., Adler C.H., Deuschl G. (2015). MDS clinical diagnostic criteria for Parkinson’s disease. Mov. Disord..

[12] Techno-Aids association: classification code for welfare equipment (CCTA95). http://www.techno-aids.or.jp/system/ (Available from: April 23, 2022).

[13] van der Marck M.A., M., Ph. C. Klok, M. S. Okun, N. Giladi, M. Munneke, B. R. Bloem, N.F.T. Force, (2014). Consensus-based clinical practice recommendations for the examination and management of falls in patients with Parkinson’s disease. Parkinsonism Relat. Disord..

[14] Tomlinson C.L., Stowe R., Patel S., Rick C., Gray R., Clarke C.E. (2010). Systematic review of levodopa dose equivalency reporting in Parkinson’s disease. Mov. Disord..

[15] Giladi N., Shabtai H., Simon E.S., Biran S., Tal J., Korczyn A.D. (2000). Construction of freezing of gait in questionnaire for patients with parkinsonism. Parkinsonism Relat. Disord..

[16] Gibson M.J. (1990). Improving the Health of Older People: a World View.

[17] I. Nara (Supervisor), Y. Matsuo and M. Ishii, . (2020). Parkinson’s Disease Physical Therapy.

[18] Jonsson L. (2001).

[19] Nilsson M.H., Drake A.M., Hagell P. (2010). Assessment of fall-related self-efficacy and activity avoidance in people with Parkinson’s disease. BMC Geriatr..

[20] Marsili L., Mahajan A. (2022). Clinical milestones in Parkinson’s disease: past, present, and future. J. Neurol. Sci..

[21] Goldstein D.S. (2006). Orthostatic hypotension as an early finding in Parkinson’s disease. Clin. Auton. Res..

[22] De Pablo-Fernandez E.D., Tur C., Revesz T., Lees A.J., Holton J.L., Warner T.T. (2017). Association of autonomic dysfunction with disease progression and survival in Parkinson disease. JAMA Neurol..

[23] Bloem B.R., Munneke M., Carpenter M.G., Allum J.H. (2003). The impact of comorbid disease and injuries on resource use and expenditures in parkinsonism. Neurology.

[24] Howland J., Peterson E.W., Levin W.C., Fried L., Pordon D., Bak S. (1993). Fear of falling among the community-dwelling elderly. J. Aging Health.

[25] Nakazono H., Matunaga K., Mihono T., Nakasima Y., Sugiyama R., Nakanisi R., Ideta T. (2012). A study on factor of recurrent falls in Parkinson’s disease from the point of view of fear of falling, General. Rehabilitation.

[26] Mirelman A., Herman T., Brozgol M., Dorfman M., Sprecher E., Schweiger A., Giladi N., Hausdorff J.M. (2012). Executive function and falls in older adults: new findings from a five-year prospective study link fall risk to cognition. PLOS ONE.

[27] Dibble L.E., Lange M. (2006). Predicting falls in individuals with Parkinson disease: a reconsideration of clinical balance measures. J. Neurol. Phys. Ther..

[28] Takakusaki K., Saito K., Habaguchi T., Ohhinata J. (2001). Regulation of gait and muscle tone by the basal ganglia. Brain Sci..

[29] Bloem B.R., Grimbergen Y.A.M., van Dijk J.G., Munneke M. (2006). The “posture second” strategy: a review of wrong priorities in Parkinson’s disease. J. Neurol. Sci..

[30] O’Shea S., Morris M.E., Iansek R. (2002). Dual task interference during gait in people with Parkinson disease: effects of motor versus cognitive secondary tasks. Phys. Ther..

